# COVID-19 Pandemic and Quality of Care and Outcomes of Acute Stroke Hospitalizations: the Paul Coverdell National Acute Stroke Program

**DOI:** 10.5888/pcd18.210130

**Published:** 2021-08-19

**Authors:** Xin Tong, Sallyann M. Coleman King, Ganesh Asaithambi, Erika Odom, Quanhe Yang, Xiaoping Yin, Robert K. Merritt

**Affiliations:** 1 Division for Heart Disease and Stroke Prevention, National Center for Chronic Disease Prevention and Health Promotion, Centers for Disease Control and Prevention, Atlanta, Georgia; 2 US Public Health Service, Atlanta, Georgia; 3 United Hospital, Allina Health, St Paul, Minnesota; 4 AllianceGroup, Inc, Atlanta, Georgia

## Abstract

**Introduction:**

Studies documented significant reductions in emergency department visits and hospitalizations for acute stroke during the COVID-19 pandemic. A limited number of studies assessed the adherence to stroke performance measures during the pandemic. We examined rates of stroke hospitalization and adherence to stroke quality-of-care measures before and during the early phase of pandemic.

**Methods:**

We identified hospitalizations with a clinical diagnosis of acute stroke or transient ischemic attack among 406 hospitals who contributed data to the Paul Coverdell National Acute Stroke Program. We used 10 performance measures to examine the effect of the pandemic on stroke quality of care. We compared data from 2 periods: pre–COVID-19 (week 11–24 in 2019) and COVID-19 (week 11–24 in 2020). We used χ^2^ tests for differences in categorical variables and the Wilcoxon–Mann–Whitney rank test or Kruskal–Wallis test for continuous variables.

**Results:**

We identified 64,461 hospitalizations. We observed a 20.2% reduction in stroke hospitalizations (from 35,851 to 28,610) from the pre–COVID-19 period to the COVID-19 period. Hospitalizations among patients aged 85 or older, women, and non-Hispanic White patients declined the most. A greater percentage of patients aged 18 to 64 were hospitalized with ischemic stroke during COVID-19 than during pre–COVID-19 (34.4% vs 32.5%, *P* < .001). Stroke severity was higher during COVID-19 than during pre–COVID-19 for both hemorrhagic stroke and ischemic stroke, and in-hospital death among patients with ischemic stroke increased from 4.3% to 5.0% (*P* = .003) during the study period. We found no differences in rates of receiving care across stroke type during the study period.

**Conclusion:**

Despite a significant reduction in stroke hospitalizations, more severe stroke among hospitalized patients, and an increase in in-hospital death during the pandemic period, we found no differences in adherence to quality of stroke care measures.

**Summary**

**What is already known on this topic?**

Studies reported significant reduction in admissions for acute stroke during the COVID-19 pandemic, but only a few studies examined the changes in stroke quality of care.

**What is added by this report?**

Using data from a multistate stroke registry funded by the Centers for Disease Control and Prevention, we found that patients with more severe strokes were admitted during the COVID-19 pandemic than during the prepandemic period, and in-hospital death rates increased. However, the adherence to stroke quality of care measurements did not change.

**What are the implications for public health practice?**

Stroke is a life-threating medical emergency; public health efforts should continue promoting awareness of stroke signs and symptoms and the urgency of seeking treatment of stroke despite the COVID-19 pandemic.

## Introduction

The US declared a national emergency in response to the COVID-19 pandemic on March 13, 2020 (1). At the same time, the Centers for Medicare and Medicaid Services (CMS) announced that patient hospitalization data from the first 6 months of 2020 would not be used in any hospital-based performance or payment programs, citing the need to focus on preparing for a potential surge of patients (2). Other quality improvement programs followed CMS recommendations (2). Since the start of the COVID-19 pandemic in the US, several studies have reported significant reductions in emergency department visits and hospitalizations for stroke (1,3–5). Stay-at-home orders, social distancing, and fear of contracting SARS-CoV-2 in health care settings might have contributed to these reductions (4,5). These reports are concerning given the established benefits of time-sensitive acute stroke treatments on long-term outcomes and lower 30-day mortality rates among patients treated in an integrated stroke care system (4). Despite multiple studies on the effect of the pandemic on stroke hospitalizations and treatment outcomes, only a few studies have assessed changes in quality of stroke care during the early phase of the COVID-19 pandemic. We used a multistate stroke registry to examine rates of stroke hospitalizations before and during the early phase of the COVID-19 pandemic as well as adherence to evidence-based performance measures for stroke hospitalizations during the pandemic.

## Methods

We used data from the Paul Coverdell National Acute Stroke Program (PCNASP), an ongoing quality improvement acute stroke program established by the Centers for Disease Control and Prevention (CDC) in 2001 to support state-based acute stroke quality-of-care registries (6). PCNASP collects de-identified data on stroke patients from participating hospitals in funded states. Case ascertainment for inclusion uses the final clinical diagnosis documented by the physician and considers the principal *International Classification of Diseases, 10th Revision, Clinical Modification* (ICD-10-CM) diagnosis codes (7). The final clinical diagnosis in PCNASP is determined by the patient’s physician and abstracted from the medical record into the hospital’s electronic data collection system. The case ascertainment and inclusion criteria for PCNASP performance measures and other analyses are based on the final clinical diagnosis only. Hospital participation in this program is voluntary. Trained abstractors used standard data definitions provided by CDC to collect detailed information on hospitalizations for stroke or transient ischemic attack (TIA) concurrent with or soon after hospitalization discharge.

We included data recorded in PCNASP on hospitalizations at 406 participating hospitals in 9 states from March 10 to June 15 in 2019 (pre–COVID-19 period), and March 8 to June 13 in 2020 (COVID-19 period), which corresponded to weeks 11–24 in both years. PCNASP data are not currently publicly available, but researchers can submit project proposals using established protocols, and CDC analysts generate data in tabular format (www.cdc.gov/dhdsp/programs/stroke_registry.htm).

Hospitalizations selected for the study period were for patients who had a clinical diagnosis of hemorrhagic stroke, including both intracerebral hemorrhage and subarachnoid hemorrhage; ischemic stroke; or TIA. We estimated the percentage reduction in hospitalizations for stroke and TIA from the pre–COVID-19 period to the COVID-19 period by age, sex, and race/ethnicity (by dividing the difference between the pre–COVID-19 and the COVID-19 periods by pre–COVID-19 hospitalizations and multiplying by 100). We used bootstrap resamples to determine 95% CIs on reduction percentages with 1,000 bootstrap resamples.

To quantify, monitor, and assess the quality of acute stroke care received, CDC in collaboration with the American Heart Association and the Joint Commission, developed 10 evidence-based performance measures (8). A patient who receives stroke care that meets all performance measures for which they are eligible is defined as receiving defect-free care (9). We examined the rates of adherence to these 10 evidence-based performance measures for acute stroke care and the percentage of patients who received defect-free care.

Demographic information collected for each hospitalized patient included age group (18–64, 65–74, 75–84, and ≥85 years), sex, race/ethnicity (non-Hispanic White, non-Hispanic Black, Hispanic, and other race (Asian, American Indian/Alaskan Native, Native Hawaiian/Other Pacific Islander, and unknown), and insurance type. Baseline clinical characteristics included 1) stroke severity upon presentation as defined by the National Institutes of Health Stroke Scale (NIHSS) score; 2) stroke onset time, defined as the time the patient was last known to be well, before the beginning of the stroke; 3) use of emergency medical services; and 4) history of hypertension, hypercholesterolemia, diabetes, myocardial infarction or coronary artery disease, atrial fibrillation, heart failure, or current tobacco use. Among patients with ischemic stroke treated with reperfusion treatments, we examined the rates of intravenous thrombolysis (IVT) and intra-arterial treatment (IAT) administered. Outcomes assessed were rates of discharge to home, in-hospital death, and hemorrhagic complications after reperfusion treatment.

We used the χ^2^ test to test for differences in distribution by demographic characteristics, clinical factors, and outcomes between the pre–COVID-19 period and the COVID-19 period. We compared continuous variables using the Wilcoxon–Mann–Whitney rank test or the Kruskal–Wallis test. To account for multiple hypothesis testing, we calculated the false-discovery rate (denoting significance by a threshold of 5%) and reported the false-discovery rate–adjusted *P* values by stroke type (10). We performed all analyses by using SAS version 9.4 (SAS Institute Inc). This study was reviewed and approved by the CDC institutional review board.

## Results

During the study period, the PCNASP identified 64,461 hospitalizations with a clinical diagnosis of hemorrhagic stroke, ischemic stroke, or TIA. From the pre–COVID-19 period to the COVID-19 period, we found an overall reduction in stroke hospitalizations of 20.2% (95% CI, 18.9%–21.3%) (Table 1) and a reduction in the number of stroke admissions from 35,851 to 28,610 (Table 2). Of reductions in the 3 types of stroke hospitalizations, the reduction among TIA hospitalizations was the largest (41.8%; 95% CI, 38.8%–44.8%), followed by ischemic stroke (18.8%; 95% CI, 17.3%–20.3%) and hemorrhagic stroke (12.4%; 95% CI, 9.0%–15.8%). For hemorrhagic stroke and ischemic stroke, but not TIA, the magnitude of reduction increased with age. Reductions in stroke hospitalization rates were greater among women than among men. By race/ethnicity, the reduction was greatest among non-Hispanic White patients and least among Hispanic patients (Table 1).

**Table 1:** Percentage Reduction of Stroke Hospitalizations Among Participating Hospitals From Weeks 11–24 in 2019 to Weeks 11–24 in 2020, by Demographic Characteristics, Paul Coverdell National Acute Stroke Program^a^

Characteristic	Percentage Reduction (95% CI)			
	All Stroke	Hemorrhagic Stroke	Ischemic Stroke	Transient Ischemic Attack
**Total**	20.2 (18.9 to 21.3)	12.4 (9.0 to 15.8)	18.8 (17.3 to 20.3)	41.8 (38.8 to 44.8)
**Age group, y**				
18–64	16.2 (14.0 to 18.3)	10.0 (4.7 to 14.8)	14.1 (11.5 to 16.9)	45.0 (39.2 to 50.4)
65–74	19.2 (16.6 to 21.6)	9.3 (2.7 to 16.7)	18.1 (16.6 to 21.6)	40.7 (34.5 to 47.0)
75–84	22.7 (20.2 to 25.1)	16.2 (8.8 to 22.9)	21.6 (20.2 to 25.1)	38.2 (31.2 to 44.1)
≥85	25.9 (23.1 to 28.9)	19.6 (11.2 to 27.6)	24.2 (20.8 to 27.1)	42.9 (35.6 to 49.4)
**Sex**				
Male	17.4 (15.5 to 19.2)	8.2 (3.1 to 13.0)	16.8 (14.7 to 18.9)	37.6 (32.8 to 42.6)
Female	23.0 (21.2 to 24.5)	16.4 (11.7 to 20.9)	20.9 (19.0 to 23.0)	45.2 (40.9 to 48.9)
**Race/ethnicity**				
Non-Hispanic White	22.3 (20.7 to 23.6)	16.4 (12.4 to 20.3)	20.7 (19.1 to 22.5)	41.2 (37.4 to 44.6)
Non-Hispanic Black	18.1 (15.1 to 21.0)	11.4 (2.9 to 19.4)	15.5 (11.7 to 19.0)	47.1 (39.7 to 53.5)
Hispanic	8.7 (2.5 to 14.9)	−2.9 (−18.0 to 10.9)	7.3 (0.1 to 14.2)	39.4 (26.5 to 51.9)
Other race^b^	15.3 (10.9 to 19.7)	3.0 (−7.5 to 13.2)	17.0 (10.8 to 22.1)	37.0 (23.5 to 48.4)

a Week 11 (March 10–16, 2019) to week 24 (June 9–15, 2019) defined as the pre-COVID-19 pandemic period and week 11 (March 8–14, 2020) to week 24 (June 7–13, 2020) as the COVID-19 pandemic weeks. Percentage reduction in number of stroke hospitalization between 2019 and 2020 is calculated as [(2019–2020)/(2019)] × 100, and the bootstrap resamples were used to determine the 95% CI with 1,000 bootstrap resamples. Data source: Paul Coverdell National Acute Stroke Program.

b Includes Asian, American Indian/Alaska Native, Native Hawaiian/Other Pacific Islander, and unknown.

**Table 2:** Demographic and Clinical Information of Acute Stroke Patients Admitted to Participating Hospitals From Weeks 11–24 in 2019 to Weeks 11–24 in 2020, Paul Coverdell National Acute Stroke Program^a^

Variable	All Stroke			Hemorrhagic Stroke			Ischemic Stroke			Transient Ischemic Attack		
	2019	2020	*P* Value^b^	2019	2020	*P* Value^b^	2019	2020	*P* Value^b^	2019	2020	*P* Value^b^
**Total**	35,851	28,610	—	5,568	4,877	—	26,543	21,557	—	3,740	2,176	—
**Age, median (IQR), y**	71 (60–81)	70 (60–81)	<.001	68 (55–79)	67 (56–78)	.21	71 (61–82)	71 (60–81)	<.001	73 (62–82)	73 (63–82)	.69
**Age group, y**												
18–64	12,189 (34.0)	10,217 (35.7)	<.001	2,414 (43.4)	2,173 (44.6)	.41	8,636 (32.5)	7,418 (34.4)	<.001	1,139 (30.5)	626 (28.8)	.51
65–74	8,814 (24.6)	7,121 (24.9)	.45	1,253 (22.5)	1,136 (23.3)	.52	6,654 (25.1)	5,447 (25.3)	.72	907 (24.3)	538 (24.7)	.93
75–84	8,521 (23.8)	6,584 (23.0)	.04	1,177 (21.1)	986 (20.2)	.43	6,394 (24.1)	5,011 (23.2)	.047	950 (25.4)	587 (27.0)	.51
≥85	6,327 (17.6)	4,688 (16.4)	<.001	724 (13.0)	582 (11.9)	.21	4,859 (18.3)	3,681 (17.1)	<.001	744 (19.9)	425 (19.5)	.93
**Male sex**	17,857 (49.8)	14,746 (51.5)	<.001	2,693 (48.4)	2,473 (50.7)	.06	13,488 (50.8)	11,228 (52.1)	.01	1,676 (44.8)	1,045 (48.0)	.12
**Race/ethnicity**												
Non-Hispanic White	24,526 (68.4)	19,059 (66.6)	<.001	3,498 (62.8)	2,923 (59.9)	.01	18,370 (69.2)	14,573 (67.6)	<.001	2,658 (71.1)	1,563 (71.8)	.93
Non-Hispanic Black	6,063 (16.9)	4,964 (17.4)	.18	929 (16.7)	823 (16.9)	.82	4,508 (17.0)	3,810 (17.7)	.07	626 (16.7)	331 (15.2)	.43
Hispanic	1,977 (5.5)	1,805 (6.3)	<.001	411 (7.4)	423 (8.7)	.06	1,348 (5.1)	1,250 (5.8)	.001	218 (5.8)	132 (6.1)	.93
Other race^c^	3,285 (9.2)	2,782 (9.7)	.03	730 (13.1)	708 (14.5)	.12	2,317 (8.7)	1,924 (8.9)	.57	238 (6.4)	150 (6.9)	.80
**Health insurance**												
Medicaid	3,525 (9.8)	2,770 (9.7)	.61	745 (13.4)	633 (13.0)	.67	2,461 (9.3)	1,968 (9.1)	.72	319 (8.5)	169 (7.8)	.69
Medicare	22,886 (63.8)	17,003 (59.4)	<.001	3,052 (54.8)	2,556 (52.4)	.06	17,342 (65.3)	12,992 (60.3)	<.001	2,492 (66.6)	1,455 (66.9)	.93
Private	7,914 (22.1)	5,563 (19.4)	<.001	1,460 (26.2)	1,090 (22.3)	<.001	5,622 (21.2)	4,084 (18.9)	<.001	832 (22.2)	389 (17.9)	<.001
Self pay/no insurance	1,180 (3.3)	959 (3.4)	.75	251 (4.5)	217 (4.4)	.89	851 (3.2)	682 (3.2)	.85	78 (2.1)	60 (2.8)	.40
Not documented	346 (1.0)	2,315 (8.1)	<.001	60 (1.1)	381 (7.8)	<.001	267 (1.0)	1,831 (8.5)	<.001	19 (0.5)	103 (4.7)	<.001
**Time between last known to be well and emergency department arrival, median (IQR), h**	4.5 (1.4–11.9)	4.9 (1.7–12.8)	<.001	4.0 (1.4–9.2)	4.1 (1.6–9.6)	.58	5.1 (1.7–12.9)	5.6 (1.9–13.8)	<.001	2.3 (1.0–6.4)	2.3 (1.0–6.4)	.69
**Arrival at hospital by emergency medical services**	15,757 (44.0)	13,471 (47.1)	<.001	2,364 (42.5)	2,085 (42.8)	.82	11,720 (44.2)	10,268 (47.6)	<.001	1,673 (44.7)	1,118 (51.4)	<.001
**NIHSS score, median (IQR)^d^**	3 (1–8)	4 (1–10)	<.001	7 (2–19)	9 (2–20)	<.001	3 (2–6)	4 (1–9)	<.001	1 (0–3)	1 (0–3)	.93
**NIHSS score^d^**												
Missing	3,885 (10.8)	3,016 (10.5)	—	1,842 (33.1)	1,503 (30.8)	—	1,737 (6.5)	1,362 (6.3)	—	306 (8.2)	151 (6.9)	—
0–4	19,149 (53.4)	14,258 (49.8)	<.001	1,559 (28.0)	1,283 (26.3)	.14	14,652 (55.2)	11,257 (52.2)	<.001	2,938 (78.6)	1,718 (79.0)	.93
5–24	11,371 (31.7)	9,936 (34.7)	<.001	1,646 (29.6)	1,526 (31.3)	.14	9,238 (34.8)	8,108 (37.6)	<.001	487 (13.0)	302 (13.9)	.70
≥25	1,446 (4.0)	1,400 (4.9)	<.001	521 (9.4)	565 (11.6)	.001	916 (3.5)	830 (3.9)	.03	9 (0.2)	5 (0.2)	.93
**Medical history**												
Hypertension	27,136 (75.7)	21,311 (74.5)	<.001	3,907 (70.2)	3,352 (68.7)	.22	20,373 (76.8)	16,293 (75.6)	.005	2,856 (76.4)	1,666 (76.6)	.93
Hypercholesterolemia	17,835 (49.7)	14,271 (49.9)	.76	2,065 (37.1)	1,857 (38.1)	.49	13,733 (51.7)	11,175 (51.8)	.86	2,037 (54.5)	1,239 (56.9)	.30
Diabetes	11,908 (33.2)	9,464 (33.1)	.76	1,292 (23.2)	1,153 (23.6)	.70	9,348 (35.2)	7,576 (35.1)	.87	1,268 (33.9)	735 (33.8)	.93
Current smoker	6,288 (17.5)	5,004 (17.5)	.87	878 (15.8)	746 (15.3)	.64	4,963 (18.7)	4,003 (18.6)	.81	447 (12.0)	255 (11.7)	.93
Myocardial infarction/coronary artery disease	7,700 (21.5)	5,997 (21.0)	.16	855 (15.4)	735 (15.1)	.77	5,855 (22.4)	4,210 (21.8)	.30	909 (24.3)	540 (24.8)	.93
Atrial fibrillation	6,549 (18.3)	5,044 (17.6)	.05	822 (14.8)	752 (15.4)	.52	5,089 (19.2)	3,875 (18.0)	.002	638 (17.1)	417 (19.2)	.23
Heart failure	3,660 (10.2)	3,026 (10.6)	.17	423 (7.6)	389 (8.0)	.63	2,854 (10.8)	2,422 (11.2)	.13	383 (10.2)	215 (9.9)	.93

Abbreviation: —, does not apply; IQR, interquartile range; NIHSS, National Institutes of Health Stroke Scale.

a Week 11 (March 10–16, 2019) through week 24 (June 9–15, 2019) defined as the pre-COVID-19 pandemic period and week 11 (March 8–14, 2020) through week 24 (June 7–13, 2020) as the COVID-19 pandemic weeks. All values are number (percentage) unless otherwise indicated. Data source: Paul Coverdell National Acute Stroke Program.

b False discovery rate-adjusted *P* values at threshold of 5%.

c Includes Asian, Native Hawaiian/Other Pacific Islander, American Indian/Alaska Native, and unknown.

d NIHSS score ranges from 0 to 42; the higher the score, the greater the impairment.

Among ischemic stroke hospitalizations, the overall percentage of patients aged 18 to 64 years significantly increased during the study period, from 32.5% 34.4% (*P* < .001) (Table 2). The percentage of patients arriving to the hospital by emergency medical services significantly increased for TIA and ischemic stroke during the study period (*P* < .001 for both) but was stable for hemorrhagic stroke (*P* = .82). Overall, the median time from stroke onset to emergency department arrival increased significantly during the study period (*P* < .001). The median NIHSS score at presentation was significantly higher during the COVID-19 period than the pre–COVID-19 period for hemorrhagic stroke and ischemic stroke, but not for TIA. We found no significant differences in medical comorbidities between the 2 periods except for hypertension and atrial fibrillation among ischemic stroke hospitalizations.

We found no differences in adherence to performance of stroke care measures among hemorrhagic stroke, ischemic stroke, and TIA hospitalizations from the pre–COVID-19 period to COVID-19 period (Table 3). Rates of defect-free care did not differ significantly by stroke type (hemorrhagic stroke, *P* = .56; ischemic stroke, *P* = .83; TIA, *P* = .79).

**Table 3:** Stroke Performance Measures, Treatments, and Outcomes, by Stroke Type Among Stroke Patients Admitted to Participating Hospitals From Weeks 11–24 in 2019 to Weeks 11–24 in 2020, Paul Coverdell National Acute Stroke Program^a^

Factor	Hemorrhagic Stroke			Ischemic Stroke			Transient Ischemic Attack		
	2019	2020	*P* Value^b^	2019	2020	*P* Value^b^	2019	2020	*P* Value^b^
**Performance measures established by Paul Coverdell National Acute Stroke Program, %**									
STK-1: Venous thromboembolism prophylaxis	98.1	98.4	.56	97.5	97.5	.99	—	—	—
STK-2: Discharged on antithrombotic therapy	—	—	—	99.6	99.6	.83	98.0	98.5	.44
STK-3: Anticoagulation therapy for atrial fibrillation/flutter	—	—	—	98.0	97.7	.59	94.0	96.3	.39
STK-4: Arrival in 2 h and alteplase given in 3 h of last known to be well	—	—	—	94.3	93.5	.54	—	—	—
STK-5: Antithrombotic therapy by day 2	—	—	—	97.7	97.5	.54	98.1	97.4	.44
STK-6: Discharged on statin medication	—	—	—	98.7	98.6	.89	94.7	95.9	.39
STK-7: Dysphagia screening	84.3	85.2	.56	87.0	87.7	.12	—	—	—
STK-8: Stroke education	92.7	92.8	.93	95.3	95.3	.95	92.1	91.4	.50
STK-9: Smoking cessation counseling	97.9	98.1	.93	98.7	98.1	.23	95.7	95.7	.98
STK-10: Assessed for rehabilitation	98.7	99.0	.56	99.3	99.3	.83	—	—	—
Defect-free care	83.7	84.7	.56	81.8	82.0	.83	87.7	88.1	.79
**Treatment, %**									
Intravenous thrombolysis or intra-arterial reperfusion treatment	—	—	—	17.0	17.7	.12	—	—	—
Intravenous thrombolysis reperfusion treatment only	—	—	—	55.7	50.6	<.001	—	—	—
Intra-arterial reperfusion treatment only	—	—	—	34.1	39.8	<.001	—	—	—
Intravenous thrombolysis and intra-arterial reperfusion treatment	—	—	—	10.2	9.5	.55	—	—	—
Any complication after reperfusion therapy^c^	—	—	—	4.0	4.5	.54	—	—	—
**Among patients given intravenous thrombolysis reperfusion treatment**									
Time between last known to be well and emergency department arrival, median (IQR), min	—	—	—	68 (44–115)	70 (44–119)	.54	—	—	—
Time between last known to be well and intravenous thrombolysis administered, median (IQR), min	—	—	—	125 (89–174)	129 (89–178)	.22	—	—	—
Time between emergency department arrival and intravenous thrombolysis administered, median (IQR), min	—	—	—	49 (35–70)	50 (34–70)	.94	—	—	—
Time between emergency department arrival and intravenous thrombolysis administered ≤60 min, %	—	—	—	66.5	66.2	.95	—	—	—
Time between emergency department arrival and intravenous thrombolysis administered ≤45 min, %	—	—	—	43.6	43.4	.96	—	—	—
**Among patients given intra-arterial reperfusion treatment**									
Time between emergency department arrival and intra-arterial treatment administered, median (IQR), min	—	—	—	92 (60–132)	96 (62–139)	.12	—	—	—
**Outcomes, %**									
Discharged to home	29.4	29.7	.86	49.7	50.9	.04	83.6	84.6	.47
Discharged to hospice	6.9	8.6	.02	4.2	5.0	<.001	0.7	0.6	.79
Discharged to acute care facility	3.3	3.1	.86	2.2	2.2	.95	0.6	1.0	.40
Discharged to another health care facility	39.4	36.6	.02	38.5	35.3	<.001	13.1	11.3	.25
In-hospital death	20.7	21.4	.56	4.3	5.0	.003	0.1	0.5	.08

Abbreviation: —, does not apply; IQR, interquartile range; STK, stroke.

a Week 11 (March 10–16, 2019) to week 24 (June 9–15, 2019) defined as the pre–COVID-19 pandemic period and week 11 (March 8–14, 2020) to week 24 (June 7–13, 2020) as the COVID-19 pandemic weeks. Data source: Paul Coverdell National Acute Stroke Program.

b False discovery rate-adjusted *P* values at threshold of 5%.

c The complication of either symptomatic intracranial hemorrhage or life-threatening or serious systemic hemorrhage within 36 hours after treatment.

The overall percentage of any reperfusion treatment among ischemic stroke patients was similar during the pre–COVID-19 and COVID-19 periods (17.0% vs 17.7% *P* = .12) (Table 3). However, the percentage of IVT administered decreased from 55.7% during the pre–COVID-19 period to 50.6% during the COVID-19 period (*P* < .001). The percentage of IAT significantly increased from 34.1% during the pre–COVID-19 period to 39.8% during the COVID-19 period (*P* < .001). The rate of any hemorrhagic complications associated with reperfusion treatments did not change (4.0% to 4.5%; *P* = .54). We found no differences in time from stroke onset to emergency department arrival (*P* = .54), stroke onset to IVT administered (*P* = .22), or emergency department arrival to IVT administered (*P* = .94) from the pre–COVID-19 period to the COVID-19 period. The median time between emergency department arrival time and IAT administered time was 92 minutes during the pre-COVID-19 period and 96 minutes during the COVID-19 period (*P* = .12). The percentage of ischemic patients who received IVT within 60 minutes and within 45 minutes (*P* = .95 and *P* = .96, respectively) were not significantly different between the 2 periods (Table 3).

The percentage of patients who were discharged to home did not differ significantly between the 2 periods for patients with hemorrhagic stroke (*P* = .86) or TIA (*P* = .47), but a significantly higher proportion of patients with ischemic stroke were discharged to home during the COVID-19 period than during the pre–COVID-19 period (50.9% vs 49.7%; *P* = .04). The rate of in-hospital death was significantly higher during the COVID-19 period than during the pre–COVID-19 period for ischemic stroke hospitalizations (5.0% vs 4.3%, *P* = .003).

## Discussion

We observed an overall reduction of 20.2% in stroke and TIA hospitalizations when we compared the pre–COVID-19 period and the early phase of the COVID-19 pandemic. The largest reduction was 41.8% for TIA, followed by ischemic stroke and hemorrhagic stroke. Hospitalization rates among stroke patients aged 85 or older, women, and non-Hispanic White patients declined the most during the pandemic. Despite changes in the volume of stroke hospitalizations and the need to focus hospital resources on the pandemic, the adherence to stroke quality of care measures did not change during the early phase of the COVID-19 pandemic among PCNASP hospitals.

The reduction in stroke hospitalizations that we observed is consistent with several studies in the US and other countries (1,3–5,11–13). Strict instructions to stay at home, the practice of social distancing, and fears of infection in medical facilities may explain the decrease in stroke hospitalizations during the early phase of the COVID-19 pandemic (12,13). Czeisler and colleagues estimated that 41% of US adults delayed or avoided medical care during the pandemic because of concerns about COVID-19 (14). Our study observed a reduction in stroke hospitalizations that increased with age, with patients aged 85 or older having the largest reduction in hospitalizations, at 25.9%. This observation is consistent with studies suggesting that older adults (aged ≥65), especially those living alone or with limited caregiver support, were more likely than younger adults to experience delays in stroke diagnosis and initiation of treatment during the COVID-19 pandemic (13,14).

TIA hospitalizations decreased by more than 40% from the pre–COVID-19 period to the COVID-19 period. This decrease may have been due to the reluctance of patients with mild stroke symptoms to seek hospital care, for fear of being exposed to COVID-19 (15). In addition, patients with minor stroke symptoms may not have sought care, or may have delayed seeking care, because of the social distancing mandates and stay-at-home orders implemented across the US (16). Without timely intervention and treatment, even for mild stroke symptoms, the risk of more severe outcomes or recurrent stroke increases (14,17,18). In our study, we found significantly higher median NIHSS scores among hospitalized stroke patients during the COVID-19 period, and the percentage of in-hospital deaths among patients with ischemic stroke significantly increased from 4.3% during the pre–COVID-19 period to 5.0% during the COVID-19 period. This finding was consistent with previous studies reporting that the decline in the number of patients admitted with mild strokes was far greater than was seen for moderate or severe strokes during the COVID-19 pandemic (17).

Rates of defect-free care in PCNASP-participating hospitals did not change during the study period across all stroke types. Specifically, the rate of stroke education delivery was not affected by the pandemic. Provision of stroke education to patients and caregivers is a critical performance measure. It provides the ideal transition from hospital to the next phase of care, and it has been shown to reduce the risk of recurrent stroke and decrease health costs for patients (9). In a recent publication, we reported that defect-free care significantly improved among patients with hemorrhagic stroke, ischemic stroke, and TIA from 2008 to 2018 among PCNASP-participating hospitals, reflecting the continuous efforts and the implementation of stroke quality improvement activities to improve the system of stroke care (9). The 10 performance measures endorsed by the American Heart Association, the Joint Commission, and CDC are essential in ensuring the quality of stroke care received by patients. Despite suspension of reporting requirements by CMS and other quality improvement programs at the beginning of the COVID-19 pandemic (2), PCNASP as a federally funded quality program led by state health departments in collaboration with the American Heart Association and emergency medical service agencies continued its quality assessments during the COVID-19 pandemic.

Although the overall rates of any reperfusion therapies among patients with ischemic stroke did not change during our study period, the use of IAT only increased significantly, and the use of IVT only decreased significantly. This reduction in IVT was likely related to the longer median time between stroke onset and emergency department arrival found among patients with ischemic stroke. This finding is consistent with other findings that indicated a lower likelihood of IVT administration during the pandemic, suggesting that patients were arriving at the hospital too late to be eligible for receiving this treatment (19,20). However, among patients receiving IVT, the time between emergency department arrival and IVT administration did not change. This finding supports the evidence for efficiencies created in the emergency department despite the need to don and doff appropriate personal protective equipment during the COVID-19 pandemic (21). Given the larger time window of opportunity for being eligible for IAT (vs IVT), we were not surprised to observe a higher rate of IAT use during the COVID-19 period than during the pre-COVID period, which is consistent with reports of increasing IAT use over time (22). Furthermore, studies reported that higher rates of large vessel occlusion with coexistent COVID-19 could increase the rate of IAT use among all ischemic stroke patients, particularly among younger patients (23,24). In our study, the frequency of hemorrhagic complications associated with reperfusion treatments did not change between the 2 study periods.

Our study has several limitations. First, PCNASP is a voluntary quality improvement program that includes hospitals from selected states; therefore, the results might not be generalizable to the US. Second, registry data did not include information on the presence or absence of COVID-19 coinfections for stroke hospitalizations; consequently, we are uncertain about how COVID-19 may have affected the outcomes during the pandemic. Third, we only included the hospitals participating in PCNASP in both study periods in 2019 (pre–COVID-19) and 2020 (COVID-19), which could have contributed to selection bias. Fourth, PCNASP uses final clinical diagnosis to determine stroke hospitalizations. Some patients with principal ICD-10-CM codes for stroke or TIA may not have been included in the registry. However, a study suggested that the concordance between ICD-10-CM codes and stroke clinical diagnosis was generally high in PCNASP, so misclassification would apply to a small number of patients (25). Finally, our study compared point prevalence data (pre-COVID-19 period in 2019 vs the early phase of the COVID-19 pandemic period in 2020); it did not examine the potential effects of long-term trends in stroke hospitalizations and quality of care because of the changes in the participating hospitals in PCNASP over time. The strengths of our study include the large volume of hospitalizations from different kinds of hospitals (rural, urban, academic, nonacademic) collected during the regular delivery of stroke care and information on stroke treatments and quality of stroke care measures from multiple states.

In summary, the rate of hospitalizations was higher among younger (aged 18–64 y) stroke patients and patients with more severe clinical conditions during the early phase of the COVID-19 pandemic than during the year before. We also observed a significant reduction in the percentage of stroke hospitalizations and an increase in overall in-hospital death from the pre–COVID-19 period to the early phase of the COVID-19 period. However, adherence to stroke quality measures and defect-free care did not change from the pre–COVID-19 period to the early phase of the COVID-19 pandemic among the hospitals participating in PCNASP.

The finding of adherence to stoke quality measures during the pandemic may be attributed to state health departments’ continued outreach to the participating hospitals and their sharing of successes and strategies in well-formed stroke system-of-care partnerships. The dissemination of these strategies and experiences may support efforts to improve the system of care and promote processes that can withstand the impact of the pandemic. Finally, these findings indicate the importance of strengthening public health efforts that promote the awareness of stroke signs and symptoms and the urgency for seeking treatment of stroke, even for mild stroke symptoms.
